# Of Elections and Emeritus Memberships

**DOI:** 10.1007/s13644-013-0142-1

**Published:** 2013-10-04

**Authors:** William H. Swatos

**Affiliations:** Baylor University, Waco, TX USA

The balloting for the 2013 RRA elections has now been completed. Your voting yielded the following outcomes: Jack Marcum was elected President for a term that begins in 2015. He became Vice-President at the end of this year’s annual meeting. Mary Gautier and Melinda Lundquist Denton were elected to 4-year terms on the RRA Board of Directors. They also took office at the end of this year’s annual meeting. Conrad Hackett and Robert Woodberry were elected to the Nominating Committee and will begin their work with the election of 2014. On behalf of the RRA membership I want to congratulate all of these individuals and extend our appreciation to everyone who agreed to stand for election, as well as to Nominations Chair Elaine Howard Ecklund and those who worked with her. Finding well-qualified persons who are willing to accept nomination is not the easiest job in RRA.

On another front, the Executive Office had quite a surprising event as dues rolled in this year, inasmuch as a number of people declared themselves Emeritus members and paid Emeritus dues. Some also included generous contributions, for which we are grateful. Others did not. This matter apparently needs to be set straight formally, so that it is included in that part of our institutional printed record that goes to all members: The RRA By-Laws revision of 1995 ended the category of Emeritus membership, but the Board also recognized at that time that this honor had been bestowed on a number of individuals and stated that those persons who held this status prior to 1995 would retain it for the remainder of their active lives in the RRA. The only RRA members who now hold this status are David O. Moberg and Everett L. Perry. We wish them both long and productive lives, but once they have gone to their rewards, that is the end of Emeritus membership in the RRA. In the future, these Emeritus members will be sent separate dues notices to avoid this confusion.

